# Applying an Extended UTAUT2 Model to Explain User Acceptance of Lifestyle and Therapy Mobile Health Apps: Survey Study

**DOI:** 10.2196/27095

**Published:** 2022-01-18

**Authors:** Eva-Maria Schomakers, Chantal Lidynia, Luisa Sophie Vervier, André Calero Valdez, Martina Ziefle

**Affiliations:** 1 Human-Computer Interaction Center RWTH Aachen University Aachen Germany

**Keywords:** technology acceptance, UTAUT2, mHealth, privacy concerns, trust

## Abstract

**Background:**

Mobile health (mHealth) care apps are a promising technology to monitor and control health individually and cost-effectively with a technology that is widely used, affordable, and ubiquitous in many people’s lives. Download statistics show that *lifestyle apps* are widely used by young and healthy users to improve fitness, nutrition, and more. While this is an important aspect for the prevention of future chronic diseases, the burdened health care systems worldwide may directly profit from the use of *therapy apps* by those patients already in need of medical treatment and monitoring.

**Objective:**

We aimed to compare the factors influencing the acceptance of lifestyle and therapy apps to better understand what drives and hinders the use of mHealth apps.

**Methods:**

We applied the established unified theory of acceptance and use of technology 2 (UTAUT2) technology acceptance model to evaluate mHealth apps via an online questionnaire with 707 German participants. Moreover, trust and privacy concerns were added to the model and, in a between-subject study design, the influence of these predictors on behavioral intention to use apps was compared between lifestyle and therapy apps.

**Results:**

The results show that the model only weakly predicted the intention to use mHealth apps (*R*^2^=0.019). Only hedonic motivation was a significant predictor of behavioral intentions regarding both app types, as determined by path coefficients of the model (lifestyle: 0.196, *P*=.004; therapy: 0.344, *P*<.001). Habit influenced the behavioral intention to use lifestyle apps (0.272, *P*<.001), while social influence (0.185, *P*<.001) and trust (0.273, *P*<.001) predicted the intention to use therapy apps. A further exploratory correlation analysis of the relationship between user factors on behavioral intention was calculated. Health app familiarity showed the strongest correlation to the intention to use (*r*=0.469, *P*<.001), stressing the importance of experience. Also, age (*r*=–0.15, *P*=.004), gender (*r*=–0.075, *P*=.048), education level (*r*=0.088, *P*=.02), app familiarity (*r*=0.142, *P*=.007), digital health literacy (*r*=0.215, *P*<.001), privacy disposition (*r*=–0.194, *P*>.001), and the propensity to trust apps (*r*=0.191, *P*>.001) correlated weakly with behavioral intention to use mHealth apps.

**Conclusions:**

The results indicate that, rather than by utilitarian factors like usefulness, mHealth app acceptance is influenced by emotional factors like hedonic motivation and partly by habit, social influence, and trust. Overall, the findings give evidence that for the health care context, new and extended acceptance models need to be developed with an integration of user diversity, especially individuals’ prior experience with apps and mHealth.

## Introduction

### Overview

Due to their affordability and ubiquity in people’s everyday lives [1], smartphone apps offer the opportunity to monitor and control health more individually and cost-effectively than ever before [2]. Mobile health (mHealth) care apps are seen as a promising technology that has the potential to improve people’s health in general; specifically, they can, for example, enhance the independence of chronically ill people [3,4] and improve rehabilitation success [5] or outcomes of diabetes self-management [6].

mHealth apps encompass a variety of health-related services (eg, support of diagnostics and treatment), tracking of infection processes (eg, contact tracing during the COVID-19 pandemic), remote monitoring, and medicine intake reminders [7]. In addition to enhancing quality of life and supporting medical therapy, mHealth apps also support chronic disease prevention (eg, with nutrition and activity monitoring), as a sedentary lifestyle is one of the key problems of our societies that lead to an increase in chronic disease prevalence [8].

Users’ technology acceptance is one decisive factor for the adoption and widespread use of technologies, including mHealth apps, but it can also be a barrier if the diverse requirements of the potential users are not understood [9]. Established technology acceptance models, such as the unified theory of acceptance and use of technology 2 (UTAUT2) [10], structure important predictive factors for the intention to use mHealth apps. However, these established models have been criticized as not being fully applicable to the health care context, as relevant factors are missing [11,12].

This research aims to improve the understanding of users’ decisions to use, or reject the use of, mHealth apps. To do so, we have built on and extended the established and widely used UTAUT2 acceptance model. As mHealth apps are essentially based on the collection and analysis—and often also transmission—of user data, privacy concerns can be a reason for rejection. Medical data are perceived as very sensitive and, thus, even more reluctantly disclosed [13]. Also, trust or distrust in the reliability and competence of technologies is an important predictor for their use [14]. Still, these factors are missing from established and widely used technology acceptance models, like the UTAUT2. Therefore, we integrated both privacy concerns and trust in the mHealth app into the UTAUT2 model and empirically tested their impact on mHealth app acceptance.

Moreover, we tested for differences in acceptance patterns between two types of mHealth apps (research question 1): currently, therapy apps targeted at existing illnesses and ailments are far less often used than lifestyle apps that, for example, should improve fitness and prevent health problems [15]. Therefore, the question arises as to whether there are differences between the factors shaping the acceptance of therapy apps compared to lifestyle apps. To study these differences, we employed our extended UTAUT2 model to compare the acceptance of therapy and lifestyle apps.

Another important research duty regarding the acceptance of mHealth apps is the integration of effects of user diversity on technology acceptance. While demographic factors, such as age, have already been a focus of research (eg, Deng et al [14], Risch [15], and Guo et al [16]), the influence of experience, digital health literacy, and personal dispositions still needs to be further understood. While we did not extensively study the impact of these factors, we nevertheless considered their importance and exploratorily analyzed their relationship to mHealth acceptance to lay a basis for future research (research question 2).

**Figure 1:**
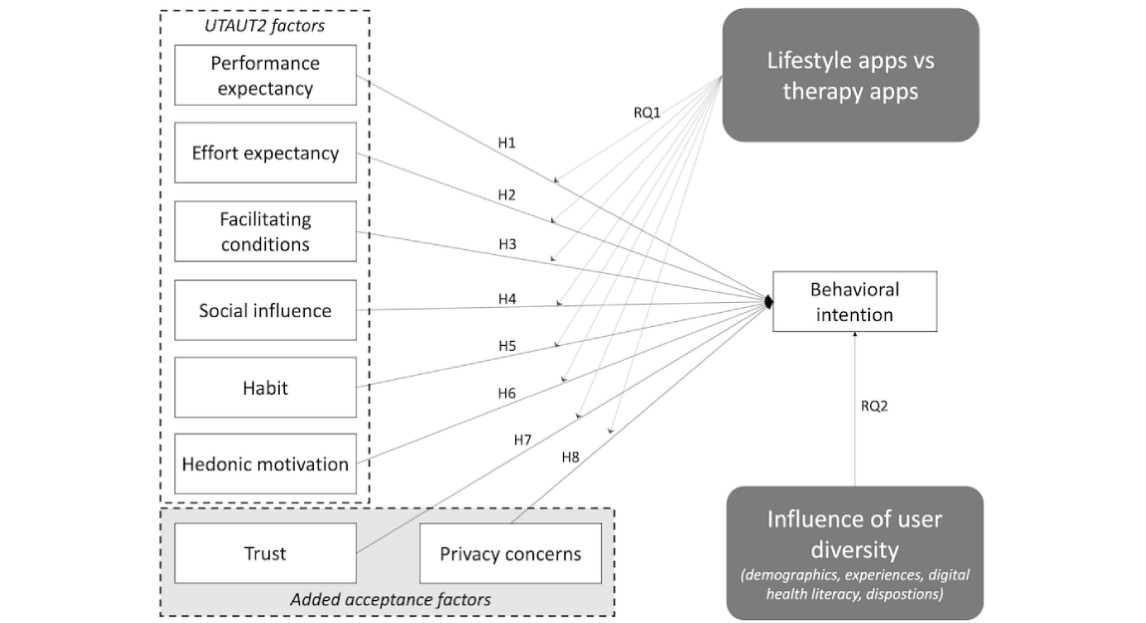
Proposed research model. H: hypothesis; RQ: research question; UTAUT2: unified theory of acceptance and use of technology 2.

Figure 1 depicts our research model, based on the UTAUT2 with the inclusion of privacy concerns and trust. The hypotheses and research questions will be developed and explained in the following sections.

Our research provides new insights into the individual and context-specific acceptance patterns for mHealth apps. This is important for revealing drivers to build acceptance as well as for identifying barriers that need to be reduced. User acceptance is one of the keys to a successful mHealth app rollout and to harnessing the full potential of mHealth for health care systems and for the improvement of quality of life and therapy for patients.

### The UTAUT2 as a Theoretical Framework

With the ever-increasing use of technology, the acceptance of new devices and software has advanced as an important focus of research. To understand the future use of new implementations, it is necessary to understand what factors influence human behavior. Based on psychological theories (eg, the theory of reasoned action [17]), technology acceptance models argue that the actual use of technology is largely influenced by a previous intention to use it, thus justifying the inclusion of not only current users but also potential future users into acceptance studies.

The most recent extension in the line of technology acceptance models is the UTAUT2 [10], which focuses on the acceptance of commercially available technologies. The UTAUT2 is often cited and has been applied and extended a multitude of times in various technology contexts (see recent reviews of the UTAUT2 [18,19]). In addition to performance expectancy, effort expectancy, social influence, and facilitating conditions, which were already part of the predecessor, UTAUT [20], in the context of private use of information technologies, factors such as fun (ie, hedonic motivation), finances (ie, price value), and habit were found to influence the behavioral intention to use technology and, thus, acceptance.

### UTAUT2 Constructs

Performance expectancy describes the perception that using the technology will provide benefits to the user and is, thus, tied to the perception of usefulness [10]. A recent review and weight analysis of UTAUT2 studies [19] showed that, indeed, in the large majority of studies, performance expectancy significantly influenced use intention and was also often the strongest predictor. Further, in mHealth research specifically, performance expectancy was continuously shown to have a significant impact on use intentions [21-26]. This applies to studies regarding lifestyle apps (eg, Schomakers et al [21] and Yuan et al [23]), therapy apps (eg, Schomakers et al [21] and Hoque and Sorwar [24]), as well as mHealth apps in general (eg, Sun et al [22] and Salgado et al [26]).

Based on this body of research, hypothesis 1 is as follows: Performance expectancy influences the intention to use mHealth apps.

Effort expectancy describes the expected ease of using the technology [10]. Results regarding the influence of effort expectancy on use intention are mixed [19]. Hoque and Sorwar [24] found effort expectancy to be a significant predictor of mHealth acceptance by older adult users, and Wang et al [25] found it to predict the intention to use online hospital mHealth services in China. Other studies could not confirm an impact on use intention of fitness and diabetic mHealth apps [21,23] or of mHealth services in general [26].

In this study, we, therefore, again examined this relationship using hypothesis 2: Effort expectancy influences the intention to use mHealth apps.

Social influence is “the degree to which an individual perceives that important others believe he or she should use the new system” [20]. Results on the significance of its influence on use intention are mixed, as approximately half of the studies applying the UTAUT2 found a significant effect [19] of social influence on acceptance. In the mHealth context, Schomakers et al [21] found social influence to be significant for both diabetes apps and fitness apps, but it showed a stronger influence on the use intention for diabetes apps. Regarding the use of mHealth by older adult users, Hoque and Sorwar [24] found a significant impact, but other studies could not confirm the effect in mHealth [23,25,26].

Therefore, hypothesis 3 is as follows: Social influence affects the intention to use mHealth apps.

Facilitating conditions refer to the perceptions “of the resources and support available to perform a behavior” [10]. For the use of mHealth apps, this is regarding, for example, a smart device on which to use apps or peers to ask about problems interacting with apps. Again, previous results are also mixed depending on the technology researched [19]. Regarding mHealth, only Wang et al [25] and Sun et al [22] found significant effects on use intention.

In this regard, we put forward hypothesis 4: Facilitating conditions influence the intention to use mHealth apps.

Hedonic motivation is the gratification counterpart to the utilitarian measure of usefulness represented by performance expectancy. Hedonic motivation refers to fun, pleasure, and enjoyment with the use of technology [10]. It is a rather strong predictor in many studies applying the UTAUT2 model [19]. In mHealth, Yuan et al [23] found a moderate effect on the intention to use fitness apps, and in a qualitative study, Woldeyohannes and Ngwenyama [27] also found evidence for its importance on mHealth app acceptance. However, Salgado et al [26] could not confirm this for the general use of mHealth.

Considering these mixed results, we examined the following relationship as hypothesis 5: Hedonic motivation influences the intention to use mHealth apps.

Habit is operationalized within the UTAUT2 framework as a self-reported perception of a customary use of the respective technology [10]. Castanha et al [19] found in their review of UTAUT2 studies that habit had good predictive abilities for use intention, and almost all studies that included habit confirmed its impact. Salgado et al [26] found habit to be the strongest predictor for mHealth use intention and found it to be the only significant one from the UTAUT2 model besides performance expectancy. The impact of habit was also confirmed for fitness apps [23]. Still, habit only has relevance for those people who already use mHealth apps.

Therefore, in this study, we only assessed habit for current users of the mHealth apps in question and proposed hypothesis 6: Habit influences the intention to use mHealth apps of current users of mHealth apps.

In the UTAUT2, a seventh predictor is the price value. Despite its significant effect on use intention as shown in some UTAUT2 studies [19,23], we did not integrate price value in our model, as most existing and well-known mHealth apps are free of charge. To include the effects of cost or price for such apps in the analysis runs the risk of price or cost obscuring all other acceptance factors, simply because people tend to reject or ascribe less value to things that are currently unaffordable to them [28]. However, we were interested in first identifying the interaction and relationship of the other acceptance factors. Therefore, the topic of cost was also left out of the description of the apps in our study.

As shown, a multitude of studies applied the UTAUT2 acceptance model in diverse application contexts. However, many researchers also extended and adapted the model to better fit the needs of the specific context of research [18]. The mHealth context is no exception to this [21,24-26]. Different illnesses or ailments have different actual or perceived repercussions. This can result in a stigma for having to deal with the ailment and can cause a fear of losing face if someone were to know about it, and these varying conditions can also affect different needs and perceived necessity in treating the illness, such as taking medication or undergoing physical treatments. Therefore, general acceptance models like the UTAUT2 can only be cautiously applied to the health care context, and extensions of the original predictors need to be considered [11,12].

### Additional Constructs

One major barrier to the use of digital and connected technologies is privacy concerns [29]. As mHealth apps also collect and analyze sensitive and intimate personal data, privacy concerns have been shown to be one important impediment to their acceptance [14,30,31].

Privacy can be defined as users’ rights to control the flow of personal information [32]. Many users feel that they have lost exactly this control over their personal information in their interaction with digital technologies [33]. They worry about malware, hackers, and identity theft as well as perceived privacy intrusion, secondary use of personal information, and perceived surveillance [34,35]. To understand what shapes privacy concerns and what consequences privacy concerns have, Smith et al [36] proposed the Antecedents–Privacy Concerns–Outcomes macromodel. It shows that privacy concerns are shaped by individual influences (ie, demographic differences and personality differences), experiences, awareness, and culture. Additionally, privacy concerns are also dependent on contextual factors [37].

A large body of research shows that privacy concerns negatively influence users’ intention to provide information [36,38]. Correspondingly, privacy concerns represent a barrier to the adoption of technologies that need personal data [29]. However, widely-used technology acceptance models, like the UTAUT2, have not yet integrated privacy concerns, and no new models that integrate privacy concerns have been established. In some empirical studies, privacy concerns were added to the established models, and could improve the prediction of acceptance in different contexts (eg, mobile banking [39], smart city technologies [40], and e-commerce [41]). Also, regarding health information technologies in general and mHealth, privacy concerns have been identified as an important factor to extend established acceptance models in qualitative [12,42,43] and quantitative [16,27,29,44,45] empirical research.

Based on these empirical results, we proposed hypothesis 7: Privacy concerns influence the intention to use mHealth apps.

Another important factor for the acceptance of information technologies and mHealth is trust [14,46,47]. Trust comes into effect in situations of uncertainty and can be described as the attitude to accept this uncertainty and vulnerability based on positive expectations [48,49]. The level of trust is based on the perceived trustworthiness of the technology [50], which is shaped by the reliability and predictability of the technology, the perceived intention of the developers, as well as the individual familiarity with the system [51].

In line with research on privacy concerns and acceptance, it could be shown that technology acceptance models should be extended by trust as an influencing factor, for example, regarding travel apps [52], mobile banking [39,53], and e-commerce [41]. For mHealth, trust has already been identified as an important extension to technology acceptance models [14,27].

This leads to our last hypothesis, hypothesis 8: Trust in mHealth apps influences the intention to use mHealth apps.

### Differentiation of Lifestyle and Therapy Apps

The available spectrum of mHealth apps is very broad. Apps related to a healthy lifestyle (eg, fitness, diet, and sleep-monitoring apps)—further on called *lifestyle apps*—are already frequently downloaded, especially by younger people [54,55]. Inspired by the “quantified self” movement and a general public awareness for the responsibility of health-conscious life and behavioral habits [56], many people are interested in using such lifestyle apps. Considering that a healthy lifestyle, regular exercise, a healthy diet, and sufficient sleep can prevent diseases, it is very welcome that mHealth apps trigger lively interest and use among end users. However, to relieve the burden on the health care system, it is necessary that all types of health service apps are offered and used; this includes lifestyle apps as well as apps related to the management of chronic and acute health conditions, the monitoring of important parameters, control of and reminders for medication intake, and other processes supporting the treatment of, and life with, chronic and acute physical or psychological conditions. These are herein called *therapy apps* and are available for different health conditions, ranging from, for example, blood pressure diaries over diabetes trackers to depression and anxiety relief.

Even though the market for lifestyle apps is twice as big as for health care apps and therapeutically orientated apps [57], mHealth apps will support the health care system if they are used by large segments of the population. Specifically regarding therapy apps, positive improvements due to the use of mHealth could be observed, for example, for the cardiac rehabilitation process in older adults [5] or the self-management of diabetes and hypertension [6]. So far, however, the quality of mHealth apps, as well as users’ intentions to use such systems, is still questionable [58].

Using apps for a general healthy lifestyle may be influenced by different motives and barriers than using apps for therapy for existing illnesses. Initial empirical evidence shows that acceptance patterns differ between different contexts of digital health technologies [45,59] as well as for different mHealth app types [21]. To better understand user acceptance of mHealth, it is important to disentangle potentially different acceptance patterns.

For these reasons, we pose research question 1: How does the influence of the proposed factors on use intention differ between lifestyle and therapy apps?

### User Diversity

People are diverse and so are their evaluation and acceptance of technologies. Besides the highly individual perceptions of, for example, performance, privacy, or influences through peers and habit, user acceptance varies depending on users’ characteristics. In general, current users of mHealth apps are rather young, female, and highly educated [54,55], but this also varies depending on the app type. In particular, therapy apps targeted to illnesses that are more prevalent in higher age groups have different target groups. The UTAUT2 incorporates age, gender, and experiences as moderating factors on the relationship between use intention and its antecedents [10]. The effects of sociodemographic characteristics have also been confirmed in other research, for example, in that different age groups attribute varying relevance on acceptance factors [16,60].

However, sociodemographic factors, specifically age, may just be carrier variables for the underlying reasons and user characteristics. The adequate know-how of handling mHealth apps, as well as the fit between needs and target groups, are factors that might have an impact on use intention [61]. Therefore, an important factor for the use and acceptance of mHealth is also familiarity and competence with the use of apps, such as digital health literacy [54,62]. Personal dispositions, such as the individual disposition to value privacy and to trust unknown technologies, may influence mHealth acceptance and explain varying importance of trust and privacy for mHealth acceptance [51,63]. In order to advance an understanding of mHealth app acceptance, the impact of these user diversity factors needs to be further examined. As this is not the focus of this study, we complement our analysis with an exploratory examination of the user diversity factors, which does not suffice for this broad topic but may give first hints on the importance of user diversity for mHealth acceptance.

Therefore, we pose research question 2: How do diverse user characteristics like sociodemographics, experience and literacy with mHealth apps, as well as personal dispositions relate to the acceptance of mHealth apps?

## Methods

We used an online questionnaire with a between-subject design to assess the opinions and acceptance by the participants of either lifestyle or therapy apps and to evaluate our hypotheses and research questions.

### The Questionnaire

In the introduction section of the questionnaire, a brief orientation on the topic of the study was given. The respondents were also reminded of their rights and informed on how the collected data would be dealt with. We encouraged them to answer freely, as there were neither “correct” nor “incorrect” answers, and we let them know that we were only interested in their perspective on this timely topic. Respondents were also informed that participation was voluntary and that they were free to quit at any time. Before starting the questionnaire, participants gave consent to collection of their data.

In the next part of the questionnaire—cataloguing the participants’ characteristics, attitudes, and experiences—their state of health was first assessed. Besides their subjective health status, questions regarding their experiences with different types of health problems were asked (ie, back and joint pain, headaches and migraine, cardiovascular diseases, allergies and food intolerances, metabolic illnesses, dementia, and a non-option). To measure their experiences with apps, the participants indicated their experience with and use of apps in general and with mHealth apps. For the use of digital health apps in particular, users require skills to search, select, appraise, and apply online health information. Therefore, digital health literacy was assessed in the next part of the questionnaire using the instrument by Van Der Vaart and Drossaert [64], which measures operational skills, navigation skills, information searching, evaluating reliability, determining relevance, adding self-generated content, and protecting privacy (see Table 1 [10,34,51,63,64] for an overview of all constructs). Concerning personality and dispositions, the personal disposition to value privacy [63] as well as the propensity to trust [51], the latter of which was adapted to apps, were assessed. At the end of the questionnaire, sociodemographics (ie, age, gender, and education level) were surveyed.

**Table 1:** Constructs used in the questionnaire with their respective sources.

Constructs	Subconstructs	Source upon which the construct was based
UTAUT2^a^ constructs	Performance expectancy Effort expectancy Social influence Facilitating conditions Hedonic motivation Habit (only answered by users) Behavioral intention (for users) Behavioral intention (for nonusers)b	Venkatesh et al [10]
Perceived trust	N/A^c^	Körber [51]
Information privacy concerns	Perceived surveillance Perceived intrusion Secondary use of personal information	Xu et al [34]
Digital health literacy	Operational skills Navigation skills Information searching Evaluating reliability Determining relevance Adding self-generated content Protecting privacy	Van Der Vaart and Drossaert [64]
Disposition to value privacy	N/A	Xu et al [63]
Propensity to trust (adapted to apps)	N/A	Körber [51]

^a^UTAUT2: unified theory of acceptance and use of technology 2.

^b^This construct was adapted from Venkatesh et al [10].

^c^N/A: not applicable; the construct in this row did not have any subconstructs.

In the main part of the questionnaire, participants were randomly assigned to evaluate either lifestyle apps or therapy apps. The evaluation started with introducing the respective mHealth apps. We assessed performance expectancy, effort expectancy, social influence, facilitating conditions, hedonic motivation, habit, and behavioral intention using the items by Venkatesh et al [10] with small adaptations to the context (a detailed overview of the items used, translations, and original items is given in Multimedia Appendix 1).

To assess privacy perceptions, the Mobile Users’ Concerns for Information Privacy [34] instrument, which consists of the three subdimensions of perceived surveillance, perceived intrusion, and secondary use of personal information, was used and adapted to lifestyle and therapy apps. To measure trust in the respective mHealth apps, items from the subdimensions of reliability and competence as well as trust in automation by Körber [51] were applied and adapted to the context.

All items were assessed on 6-point symmetric Likert scales ranging from 1 (low agreement) to 6 (strong agreement). Items were randomized to prevent biases. The language of the questionnaire was German, as only German participants were recruited; therefore, items were translated into German. For trust, no validated German translation was available. Therefore, the items were forward-translated by a German native speaker and, to test the comprehensibility and correct translations, two authors translated these again back into English. The results were compared to the original items, and deviations were settled in a discussion. During the translation process, we also adapted the items to the mHealth context and discussed these adaptations.

To assure a realistic and empathetic evaluation of mHealth apps, the formulation of the items was, on the one hand, individually adapted to the participants already being users of such apps or nonusers. For example, one item for behavioral intention was modified in the following way: “I intend to continue using such a [medical or lifestyle] app” for users versus “I intend to use such a [medical or lifestyle] app in the future” for nonusers. Also, only current and prior users were asked to answer questions concerning habit. Moreover, as therapy apps are less widespread and, therefore, less familiar, the description of therapy apps was illustrated by an example of apps that matched the participants’ experienced health problems, as in the questionnaire section about state of health. For those participants who had not experienced any of these health problems before, a general description with several examples was used.

Before distributing the study, we pretested the questionnaire and participants reported back on comprehensibility issues. Only after those issues had been eliminated did we start the data acquisition.

### Recruitment of the Sample

Participants were recruited from a university seminar and its attendees’ social contacts. Participants accessed the questionnaire via a weblink that was given to them. The comparison between the app types used a between-subject design. Thus, each participant either answered the items regarding therapy apps or lifestyle apps. The app type to be evaluated was assigned randomly. The participants volunteered to take part in the study and were not rewarded for their efforts. Data were collected in May and June of 2019.

The recruitment method was chosen with the aim to reach mHealth users as well as nonusers of therapy and lifestyle apps. Additionally, participants of different age groups were recruited. However, in accordance with the technical requirements of mHealth use, only participants with access to the internet and digital devices were targeted. Today, young people, in particular, use mHealth apps [54]. Therefore, an additional aim for recruitment was to reach those people who have the technological access and know-how to use mHealth apps but still have not adopted this technology, despite possible medical conditions (eg, relatives or acquaintances of seminar students).

### Data Analysis

The following sections will detail the analysis methods as well as regulations we applied to our data.

#### Item Analysis

We checked reliability by using Cronbach α and applied a threshold of a>.70 for all scales not included in the structural model (ie, disposition to value privacy, propensity to trust apps, and digital health literacy). Additionally, as some of the translated German items were not validated previously, we conducted an exploratory factor analysis on the model constructs, confirming the validity of the items (Multimedia Appendix 2).

#### Structural Model

Our research model was tested using partial least squares (PLS) structural equation modeling (SEM). PLS is a component-based SEM method that is suitable for exploratively testing new models [65], such as this extension of the validated UTAUT2. The analysis process was divided into two parts. First, the measurement quality was checked for reliability and validity. Only when the quality of the model was confirmed, the structural model was analyzed and interpreted.

The software SmartPLS (version 3.3) was used for the SEM modeling [66]. It offers the possibility to conduct multigroup analysis (MGA) to test differences in relationships between constructs and between user groups. We used MGA to test differences between the two app types regarding the strength of the relationships.

As we tailored the questionnaire distinctly, using the targeted app examples, we had to ensure that this did not introduce a systematic error between different apps. We first checked whether the correlations between behavioral intention and the predictor variables differed significantly between the eight app examples used. No such differences were prevalent so that, in the final analysis, no differentiation was made between the participants evaluating therapy apps with different health or ailment foci. For all analyses, a significance level of 5% was set.

#### Correlation Analysis

To describe how demographics and other user characteristics might be associated with our model variables, we used correlation analysis. To deal with suboptimal normality of our data, we used bias-corrected and accelerated bootstrapping [67].

#### Exclusion of Participants

Of 951 people who started the questionnaire, 799 completed it (84.0% completion rate). Further, 92 participants with a response time shorter than 50% of the median response time (<16 minutes, 17 seconds) were labeled as speeders and excluded. Finally, 707 participants were included in the analysis.

### Data Availability

Access to the anonymized data set can be requested on the Open Science Framework repository [68].

## Results

### The Sample

The demographic characteristics of the sample are depicted in Table 2 and are differentiated by the type of app the participants evaluated. All in all, the sample included German participants between the ages of 16 and 89 years (mean 36.8, SD 17.5) and 428 women out of 707 (60.5%). The demographic characteristics of the participants were evenly distributed between the two app types. Most participants (n=517, 73.1%) possessed a high education level with a general qualification for university entrance.

**Table 2:** Demographic characteristics of the sample comparing participants evaluating lifestyle apps and therapy apps (N=707).

Characteristic			Participants evaluating lifestyle apps (n=355)		Participants evaluating therapy apps (n=352)
Age (years), mean (SD)			36.4 (18.1)		37.3 (16.8)
**Gender, n (%)**					
	Women	222 (62.5)		206 (58.5)	
	Men	133 (37.5)		146 (41.5)	
**Education level**					
	No certificate	6 (1.7)		9 (2.6)	
	Certificate of secondary education	25 (7.0)		25 (7.1)	
	General certificate of secondary education	59 (16.6)		63 (17.9)	
	General qualification for university entrance	262 (73.8)		255 (72.4)	

Most of the 707 participants used digital technologies: 695 participants (98.3%) owned a smartphone. Only 33 participants (4.7%) did not use apps regularly. Correspondingly, the participants’ self-rated app familiarity was quite high (mean 4.32, SD 1.36), as rated on a scale from 1 (very low agreement) to 6 (very high agreement). In contrast, the familiarity with health apps was lower (mean 3.44, SD 2.56). Of the 355 participants evaluating the lifestyle mHealth apps, 110 (31.0%) were current users and 82 (23.1%) had used a lifestyle app before. Only 18 (5.1%) of the 352 participants assigned to the therapy app evaluation group were current users, and 18 (5.1%) had used a therapy app before.

Disclosing information about health status was optional in order to not be too invasive regarding the participants’ privacy. Most of the 707 participants reported their health status as “good” (n=305, 43.1%), “very good” (n=216, 30.6%), or “excellent” (n=65, 9.2%). Out of 707 participants, 5 (0.7%) reported their health status as “very bad,” 25 (3.5%) reported it as “bad,” and 83 (11.7%) reported it as “rather bad.” Out of 707 participants, 8 (1.1%) chose not to answer. Out of 707 participants, 26.9% (n=190) lived with a chronic illness, 11.9% (n=84) depended on a medical assistive device, and 31.4% (n=222) needed regular checkups with their physician.

On average, the sample showed a neutral propensity to trust apps in general (mean 3.07, SD 0.79) and a slightly stronger than neutral disposition to value privacy (mean 3.88, SD 1.14). The mean digital health literacy was quite high (mean 4.53, SD 0.75).

### The Measurement Model

To assess the quality of the measurement model, the guideline by Hair et al [65] was followed. For the reliability of the model, we confirmed internal consistency reliability (composite reliability >0.708) and considered indicator reliability (outer loading >0.7). The outer loading of one of the items for facilitating conditions on the construct was below 0.4. Dropping it improved reliability. Three other items from the constructs habit, perceived surveillance, and facilitating conditions were closely below the recommended threshold of 0.7, yet they were above 0.6, and were kept in the model as they stemmed from validated models, and dropping them decreased the reliability of the remaining model.

### The Structural Model

Evaluation of the validity included convergent validity (average variance extracted >0.5) and discriminant validity, using the Fornell-Larcker criterion. Mobile users’ information privacy concerns were modeled as higher-order models because the latent factor privacy concerns was based on three subdimensions. Therefore, validity criteria did not apply to the discriminant validity between the subdimensions themselves or between the subdimensions and the overall scale privacy concerns.

**Figure 2:**
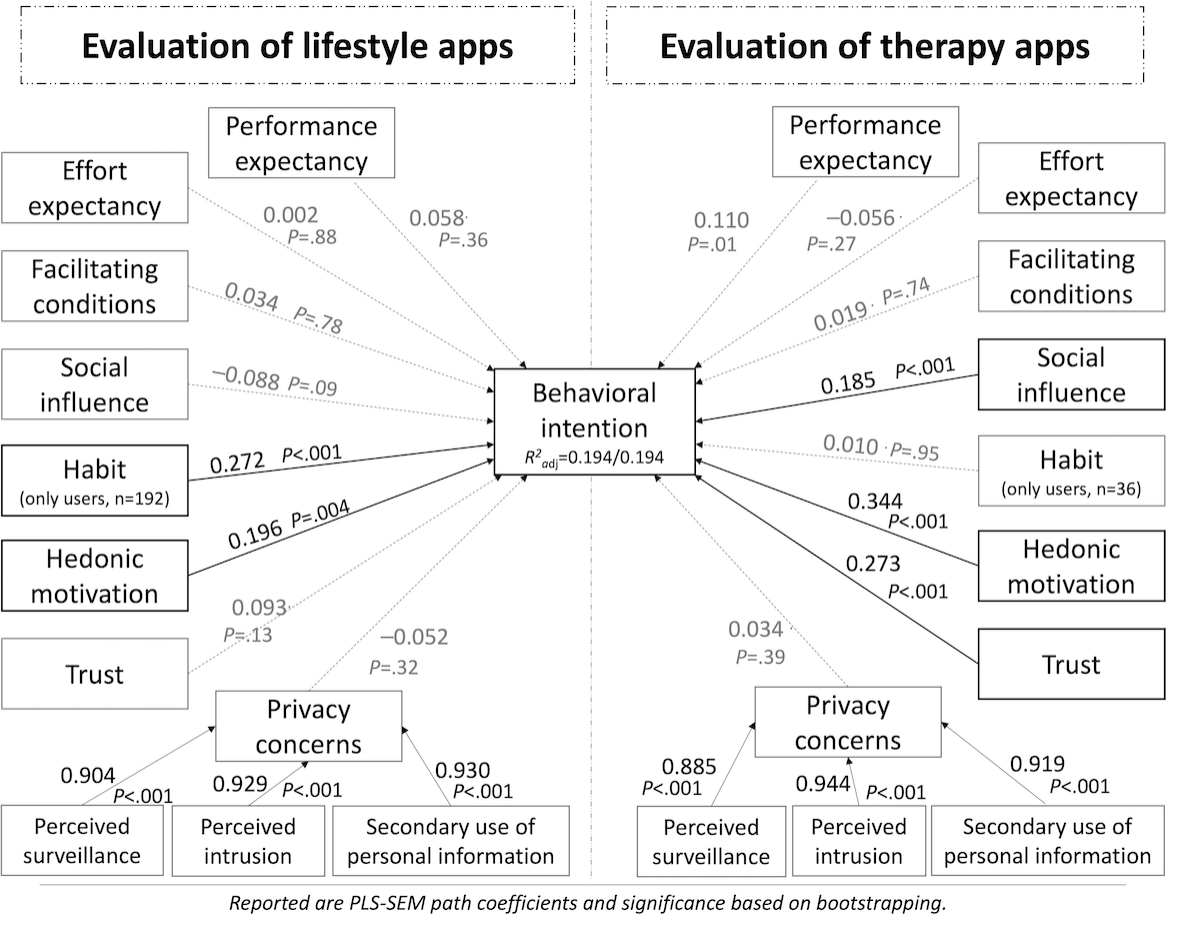
The structural model with path coefficients juxtaposed for lifestyle and therapy apps (significance based on bootstrapping; lifestyle: n=355; therapy: n=352). PLS-SEM: partial least squares structural equation modeling; adj: adjusted.

The resulting path coefficients of the model for both lifestyle and therapy apps are depicted in Figure 2. The significance of the path coefficients was checked using bias-corrected and accelerated bootstrapping with 5000 subsamples. Blindfolding procedures were calculated to assess the predictive relevance of the constructs for each app type.

The results revealed that only 19% of the variance in behavioral intention for both types of mHealth apps could be explained by the extended UTAUT2 model. The variables correspondingly showed only weak predictive relevance for behavioral intentions (Q=0.119 for both app types). Most hypothesized variables showed no significant relationship to behavioral intention. Regarding both types of apps, neither the UTAUT2 constructs of performance expectancy, effort expectancy, and facilitating conditions nor privacy concerns predicted acceptance. Hedonic motivation was the only included construct that had a significant impact on behavioral intention for both app types (lifestyle: 0.196, *P*=.004, *f^2^*=0.044; therapy: 0.344, *P*<.001*, f^2^*=0.044). Social influence did impact behavioral intention to use therapy apps (0.185, *P*<.001, *f^2^*=0.011) but not lifestyle apps. The other way around, habit did impact the intention to use lifestyle apps (0.272, *P*<.001, *f^2^*=0.02) but not therapy apps. As only current and previous users evaluated habit, the calculation was based on 192 participants for lifestyle apps and 36 participants for therapy apps. In the same vein, trust in the app showed an impact on the intention to use therapy apps (0.273, *P*<.001, *f^2^*=0.001) but not lifestyle apps.

The MGA confirmed these differences between the evaluation pattern for the app types. Significant differences were present regarding the relationships of habit (D=0.264*, P*=.002), social influence (D=0.275, *P*<.001), and trust (D=0.181, *P*=.04). Table 3 lists the bootstrapped CIs of the path coefficients and the MGA results.

**Table 3:** Bias-corrected and accelerated bootstrapped 95% CIs for the evaluation of lifestyle and therapy apps and significance of the difference in path coefficients between the two app types based on multigroup analysis (MGA).

Relationship		Lifestyle apps (n=355), 95% CI	Therapy apps (n=352), 95% CI	Significance of MGA, *P* value
**Predicting behavioral intention**				
	Performance expectancy	–0.069 to 0.185	–0.002 to 0.227	.57
	Effort expectancy	–0.100 to 0.141	–0.152 to 0.040	.41
	Facilitating conditions	–0.125 to 0.123	–0.078 to –0.093	.98
	Social influence	–0.197 to 0.013	0.089 to 0.275	<.001
	Habit	0.141 to 0.381	–0.126 to 0.106	.002
	Hedonic motivation	0.061 to 0.328	0.214 to 0.470	.12
	Trust	–0.027 to 0.214	0.146 to 0.399	.04
	Privacy concerns	–0.160 to 0.049	–0.041 to 0.110	.19
**Higher-order model of privacy concerns**				
	Perceived surveillance	0.870 to 0.951	0.850 to 0.911	.38
	Perceived intrusion	0.885 to 0.951	0.924 to 0.959	.38
	Secondary use	0.907 to 0.945	0.894 to 0.939	.51

### User Diversity in the Acceptance of mHealth Apps

The validated UTAUT2 model including the additional constructs of privacy concerns and trust showed only weak predictive relevance in explaining why people use mHealth apps and why not. The UTAUT2 postulates that age, gender, and experience moderate the relationships of the predictor variables with behavioral intention [10].

These moderators were not included in our model, as we focused on the direct relationships. Additionally, other human factors have been shown to influence the acceptance of digital technologies and mHealth. Therefore, in an exploratory attempt to decipher how user diversity influences mHealth acceptance and usage, we calculated correlations to get first hints as to what may influence behavioral intention. These results shall not represent a detailed analysis but should give first insights into the impact of selected user characteristics on the acceptance of mHealth apps.

Table 4 depicts the correlations of user factors with behavioral intention to use mHealth apps; these are not differentiated between the two app types. All variables showed significant relationships with behavioral intention. Particularly, familiarity with health apps showed a moderate effect, with a higher familiarity with health apps related to a higher intention for the ongoing use of health apps (*r*=0.469, *P*<.001). Additionally, app familiarity (*r*=0.142, *P*<.007), propensity to trust apps (*r*=0.191, *P*<.001), as well as digital health literacy (*r*=0.215, *P*<.001) increased the acceptance of health apps, showing further how important experience and familiarity are to intention for use. Also, demographic characteristics, such as age and gender, showed a significant relationship to acceptance. Older participants and men showed lower acceptance (age: *r*=–0.15, *P*<.004; gender: *r*=–0.075, *P*=.048), and participants with a higher level of education showed higher acceptance (*r*=0.195, *P*<.001). Even though privacy concerns regarding the app itself did not have an impact on behavioral intentions in the structural model, the disposition to value privacy correlated with behavioral intention (*r*=–0.194, *P*<.001). Participants who valued their privacy more showed less intention to use mHealth apps.

**Table 4:** Pearson correlation coefficients of user factors with behavioral intention to use mobile health apps with bias-corrected and accelerated 95% CIs (N=707).

User factor	Correlation with behavioral intention, *r*	95% CI	*P* value
Age	–0.150	–0.255 to –0.034	.004
Gender	–0.075	–0.152 to 0.004	.048
Education level	0.088	0.001 to 0.171	.02
App familiarity	0.142	0.054 to 0.240	.007
Health app familiarity	0.469	0.379 to 0.548	<.001
Digital health literacy	0.215	0.119 to 0.313	<.001
Privacy disposition	–0.194	–0.299 to –0.083	<.001
Propensity to trust apps	0.191	0.88 to 0.291	<.001

## Discussion

### Overview

The objective of this study was to increase the understanding of users’ acceptance and decision patterns to use mHealth apps and which factors impact acceptance for lifestyle apps compared to therapy apps. Therefore, we applied the established technology acceptance model UTAUT2 [10] to the evaluation of mHealth apps. The original model was extended with trust and privacy concerns as predictors of use intention because previous research showed their relevance in the context of digital health technologies (eg, Woldeyohannes and Ngwenyama [27], Lidynia et al [30], and Schomakers et al [59]). In an online questionnaire, 707 participants evaluated either lifestyle or therapy apps in order to compare the decision patterns between these two mHealth app types.

### Principal Findings

#### Overview

In this study, the UTAUT2 model with its extensions can only explain a small amount of variance in the intention to use mHealth apps (approximately 20%). From the original validated UTAUT2 model, only the constructs hedonic motivation, habit, and social influence partly predict the intention to use mHealth apps. The constructs effort expectancy and performance expectancy, which are similarly modeled as main aspects in other established acceptance models like the technology acceptance model [69] and the UTAUT [20], show no significant influence on acceptance. Although researchers already criticized the applicability of these established and widely used acceptance models for the health care context [11,12], our findings deviate from previous empirical research on mHealth app acceptance in which at least some of the factors proposed by the UTAUT2 influenced the intention to use mHealth apps [22,23,25,26]. Therefore, further confirmation of our results is needed, taking into account the limitations of our study, which will be further discussed hereafter, for example, regarding the sample. However, much previous research showed that, on the one hand, not all factors proposed by the UTAUT2 or other technology acceptance models had a significant impact and that, on the other hand, these need to be extended by further influencing factors [22,24,26]. This empirical evidence allows us to conclude that applicability of the UTAUT2 and its predecessors is not fully applicable to the health care and mHealth context, and extended or rather new acceptance models for this context are needed.

Also, in contrast to previous studies (eg, Guo et al [16], Bélanger and Crossler [29], and Schomakers et al [59]), privacy concerns were not found to influence the intention to use mHealth apps. Trust in the reliability and competence of the app, on the other hand, showed a small effect on the acceptance of therapy apps but not on the acceptance of lifestyle apps. Trust and privacy concerns have been extensively studied in information systems research [70-72]. However, for both concepts, no commonly agreed-upon definition and operationalization exist in research. After all, privacy and trust are not completely disjunct (eg, trust beliefs can mitigate privacy concerns) [59,73]. For both reasons, it is important to study different aspects of privacy and trust. Our results suggest that privacy concerns regarding the perceived surveillance, intrusion, and secondary use of information do not impact mHealth acceptance, but maybe concerns regarding hacker attacks, misuse of information by health insurance companies, or similar concerns do. In the same vein, trust in the reliability and competence of mHealth apps showed only a weak influence on the acceptance of therapy apps in our study. Trusting the mHealth app provider, the data protection mechanisms, or a physician recommending the use of an app may have an impact. These other dimensions of trust and privacy need to be further examined while paying close attention to the specific operationalization of the constructs. Therefore, our results are only a first step toward studying the impact of privacy and trust on mHealth acceptance.

Our results further suggest that instead of the more “utilitarian” aspects of perceived usefulness and performance of mHealth apps, it is rather the “emotional” aspects, such as fun, prior experiences, and recommendations by peers, that are important for their use. This finding must be confirmed and further analyzed in future studies, but it indicates that, on the one hand, approaches that address user experience, such as gamification, are important for mHealth apps of both types as the hedonic motivation influenced use intention for both app types. On the other hand, personal and peer experiences are very influential, whereby a widespread use of mHealth apps becomes even more important.

#### Context Differences

Besides the general model, our results revealed differences in the importance of some predictors for lifestyle and therapy apps. In our sample and in general, lifestyle apps were far more frequently used than therapy apps. The categorization of mHealth apps into lifestyle and therapy apps is not disjunct, as some apps may have functions providing both. In our study, the introduction given to the participants clearly distinguished between apps used to improve fitness, nutrition, and similar for “lifestyle” and those apps providing support for dealing with a prevailing illness. However, for future research, a classification of mHealth apps that is commonly agreed upon is vital, as is the simplification of research on context differences.

Habit emerged as a significant acceptance factor only for the lifestyle apps, which may be explained by the more widespread use and larger proportion of users in the sample. However, as the sample of users for therapy apps was very small (ie, only 36 participants), these results have to be interpreted with caution. The behavioral intention to use therapy apps was, in contrast to lifestyle apps, also influenced by social influence and trust. In this medical context, the participants need more than fun to use the app and, rather, should search for more reliable and trustworthy apps. Similar results have been found by Schomakers et al [21].

#### User Diversity

All in all, the predictive relevance of the factors in the extended UTAUT2 model is rather weak. This confirms other authors’ opinions regarding health care technologies, in that the established models can only be cautiously applied and need further adaptations, or rather, new models for the special health care context are needed (eg, Ziefle and Wilkowska [9] and Venkatesh [10]).

The results of our exploratory analysis of the relationship between different user factors and behavioral intention to use mHealth apps imply that user diversity is an important aspect that needs to be considered. In particular, experience showed a strong relationship with use intention in this preliminary analysis, particularly the experience with health apps, but also with apps in general. The same was true for digital health literacy. Further empirical research and analysis of user diversity, especially the importance of experience, is needed, but this first result hints at a developing acceptance. When more and more people use mHealth apps, including therapy apps, the increased familiarity combined with habit and social influence may increase acceptance within the population. On the other hand, it cannot be assumed that everybody has the experience and the skills to use mHealth apps. Digital health literacy has to be developed and, following the gray digital divide, older people in particular, who are also more prone to chronic conditions, need support in getting to know these digital helpers.

### Limitations

Despite the valuable insights into decision patterns regarding mHealth apps, this online questionnaire approach needs to be considered methodologically. Instead of actual adoption behavior, *reported* attitudes, perceptions, and intention to adopt were measured for mHealth apps in general, not regarding a specific mHealth app. Therefore, more general implications can be extracted from the results; however, on the other hand, users evaluated a vague idea of what mHealth apps are. Furthermore, their evaluations may be strongly influenced by those mHealth apps they have already experienced, especially as experience was shown to be strongly related to acceptance. This adds variability to the data. Therefore, this general research should be accompanied by more research into app-specific acceptance, which can provide detailed results for the optimal design of apps. Moreover, as an urgent research desideratum, the role of prior experience needs to be further explored. While the difference between users and nonusers [74] of mHealth apps might impact technology acceptance, so too could the usage of different mHealth apps probably influence future acceptance of mHealth apps in general. Additionally, the rather young and educated sample needs to be considered, which was acquired via social contacts. Convenience sampling has the advantage that those people actually participating are often highly motivated to provide their opinion. However, by their motivation and their self-selection, bias may have been brought into the data.

Besides the young and healthy persons still improving their health via lifestyle apps, thereby preventing chronic diseases, very important target groups for mHealth are older people and people with health problems. Here, mHealth can unfold its potential in directly supporting therapy and monitoring diseases, thereby improving quality of care and relieving the health care systems in a short time. These user groups should be further researched as they are underrepresented within our sample. Also, the German nationality of the participants limits the implications from this research, as attitudes toward technologies are highly influenced by cultures (eg, Trepte et al [75] and Alagöz et al [76]).

As no validated translation of the UTAUT2 items to German was known to us when planning the study, and no validated adaptation to the health care context was yet available, the use of unvalidated translations and adaptations of the items might have lowered the validity of our results. We could statistically assure a good validity and reliability of our items; nevertheless, the use of validated scales is highly recommended for future research (for a German translation of UTAUT2 items, see Harborth and Pape [77]; for a validated French adaptation to eHealth technologies, see Hayotte et al [78]).

### Conclusions

In this study, an extended UTAUT2 technology acceptance model was used to predict behavioral intention to use mHealth apps. Only a few hypothesized predictors (ie, hedonic motivation, habit, and social influence) showed a significant relationship to use intention, and the model only explained a comparably small amount of variance (approximately 20%). These factors indicate that more emotional factors than utilitarian usefulness influence mHealth app acceptance, adding a piece to the understanding of the mHealth acceptance puzzle. Small differences in the decision patterns were prevalent between the acceptance of lifestyle apps (eg, for fitness, nutrition, and sleep) and therapy apps (eg, for the monitoring and treatment of back pain, migraine, and cardiovascular diseases). In future research, the results need to be replicated, as the generalizability from our rather young sample is limited. However, our results in combination with previous research indicate that the UTAUT2 model, which was developed for the acceptance and use of mobile internet technologies in general, is not very suitable to predict mHealth use. The health care context needs improved and adapted technology acceptance models, which must also include human factors, such as experience, to account for user diversity.

## Appendix

Multimedia Appendix 1 Questionnaire items, sources, and translations.

Multimedia Appendix 2 Exploratory factor analysis.

## Abbreviations

MGA: multigroup analysis
mHealth: mobile health
PLS: partial least squares
SEM: structural equation modeling
UTAUT2: unified theory of acceptance and use of technology 2
