# Supplementary material for: Applying an Extended UTAUT2 Model to Explain User Acceptance of Lifestyle and Therapy Mobile Health Apps: Survey Study
Source: JMIR Mhealth Uhealth. 2022 Jan 18;10(1):e27095. doi: 10.2196/27095 (PMC8808343; doi:10.2196/27095)
Supplement: Multimedia Appendix 1 [file mhealth_v10i1e27095_app1.docx]

## Multimedia Appendix 1: Items, sources and translations

| **Items** [*verbatim English translation*] | Original Items (Source) |
| --- | --- |
| **Performance Expectancy** [10] | |
| Ich finde eine solche App im Alltag nützlich.  [“I find such an app useful in my daily life.“] | I find mobile Internet useful in my daily life. |
| Eine solche App hilft mir, Dinge schneller zu erledigen.  [“Using such an app helps me accomplish things more quickly.“] | Using mobile Internet helps me accomplish things more quickly. |
| Eine solche App hilft mir, besser auf meine Gesundheit zu achten.  [“Using such an app helps me take care of my health.”] | Using mobile Internet increases my productivity. |
| **Effort Expectancy** [10] | |
| Es fällt mir leicht, die Nutzung einer solchen App zu erlernen.  [“Learning how to use such an app is easy for me.”] | Learning how to use mobile Internet is easy for me. |
| Der Umgang mit einer solchen App ist klar und verständlich.  [“Interacting with such an app is clear and understandable.“] | My interaction with mobile Internet is clear and understandable. |
| Ich finde solche Apps einfach zu bedienen.  [“I find such apps easy to use.“] | I find mobile Internet easy to use. |
| Es fällt mir leicht, den Umgang mit solchen Apps zu meistern.  [“It is easy for me to become skillful at using such apps.“] | It is easy for me to become skillful at using mobile Internet. |
| **Social Influence** [10] | |
| Menschen, die mir wichtig sind, denken, dass ich eine solche App nutzen sollte.  [“People who are important to me think that I should use such an app.”] | People who are important to me think that I should use mobile Internet. |
| Menschen, die mein Verhalten beeinflussen, denken, dass ich eine solche App nutzen sollte.  [“People who influence my behavior think that I should use such an app.“] | People who influence my behavior think that I should use mobile Internet. |
| Menschen, deren Meinung ich schätze, möchten, dass ich eine solche App nutze.  [“People whose opinions I value would like me to use such an app.“] | People whose opinions that I value prefer that I use mobile Internet. |
| **Facilitating Condition** [10] | |
| Ich habe ein Smartphone oder Tablet, auf dem ich eine solche App nutzen kann.  [“I have a smartphone or tablet on which to use such an app.“] | I have the resources necessary to use mobile Internet. |
| Ich habe die notwendigen Kenntnisse, um eine solche App zu nutzen.  [“I have the necesarry knowledge to use such an app.“] | I have the knowledge necessary to use mobile Internet. |
| Ich kann Hilfe von anderen bekommen, wenn ich Schwierigkeiten bei der Nutzung einer solchen App habe.  [“I can get help from others when I have difficulties in using such an app.“] | I can get help from others when I have difficulties using mobile Internet. |
| Mein Smartphone oder Tablet hat die passende Größe, um angenehm auch längere Texte zu lesen.  [“My smartphone or tablet has the right size to read even longer texts comfortably.”] | Mobile Internet is compatible with other technologies I use. |
| **Hedonic Motivation** [10] | |
| Eine solche App zu nutzen macht Spaß.  [“Using such an app is fun.“] | Using mobile Internet is fun. |
| Eine solche App zu nutzen ist angenehm.  [“Using such an app is enjoyable.“] | Using mobile Internet is enjoyable |
| Die Nutzung einer solchen App ist sehr unterhaltsam.  [“Using such an app is very entertaining.“] | Using mobile Internet is very entertaining. |
| **Habit** (only when user) [10] | |
| Die Nutzung einer solchen App ist für mich zur Gewohnheit geworden.  [“The use of such an app has become a habit for me.”] | The use of mobile Internet has become a habit for me. |
| Ich komme ohne eine solche App nicht mehr aus.  [“I cannot do without such an app anymore.“] | I am addicted to using mobile Internet |
| Ich brauche eine solche App.  [“I need such an app.”] | I must use mobile Internet. |
| **Behavioral Intention** (users) [10] | |
| Ich beabsichtige, eine solche App weiterhin zu nutzen.  [“I intend to continue using such an app.“] | I intend to continue using mobile Internet in the future. |
| Ich werde immer versuchen, eine solche App in meinem Alltag zu nutzen.  [“I will always try to use such an app in my daily life.“] | I will always try to use mobile Internet in my daily life. |
| Ich plane mit der regelmäßigen Nutzung einer solchen App fortzufahren.  [“I plan to continue the frequent use of such an app.”] | I plan to continue to use mobile Internet frequently. |
| **Behavioral Intention** (non-users) (adapted from [10]) | |
| Ich beabsichtige, eine solche App auszuprobieren.  [“I intend to try out such an app.“] |  |
| Ich möchte eine solche App gerne in meinen Alltag integrieren.  [“I want to use such an app in my daily life.“] |  |
| In Zukunft werde ich eine solche App regelmäßig nutzen.  [“In the future I will use such an app frequently.“] |  |
| **Perceived Trust [52]** | |
| Solche Apps arbeiten zuverlässig.  ["Such apps work reliably.“] | The system works reliably. |
| Ich bin davon überzeugt, dass solche Apps gut funktionieren.  [“I am confident that such apps work well. | I am confident about the system’s capabilities. |
| Solche Apps können meinen Gesundheitszustand korrekt interpretieren.  [“Such apps are capable of interpreting my health status correctly.“] | The system is capable of interpreting situations correctly. |
| Ich vertraue Gesundheits-/Therapie-Apps.  [“I trust health/therapy apps.“] | I trust the system. |
| Ich kann mich auf Gesundheits-/Therapie-Apps verlassen.  [“I can rely on health/therapy apps.“] | I can rely on the system. |
| **Privacy Concerns [35]** |  |
| **Perceived Surveillance [35]** | |
| Ich glaube, dass der Standort meines Smartphones zumindest teilweise aufgezeichnet wird.  [“I believe that the location of my mobile device is monitored at least part of the time.“] | I believe that the location of my mobile device is monitored at least part of the time. |
| Ich bin besorgt, dass Gesundheits-/Therapie-Apps zu viele Informationen über mich sammeln.  [“I am concerned that health/therapy apps are collecting too much information about me.“] | I am concerned that mobile apps are collecting too much information about me. |
| Ich bin besorgt, dass Gesundheits-/Thearpie-Apps meine Smartphone Aktivitäten aufzeichnen.  [“I am concerned that health/therapy apps may monitor my activities on my mobile device.“] | I am concerned that mobile apps may monitor my activities on my mobile device. |
| **Perceived Intrusion [35]** | |
| Durch die Nutzung von Gesundheits-/Therapie-Apps sind persönliche Daten, die ich für mich behalten möchte, anderen Menschen leichter zugänglich, als mir lieb ist.  [“I believe that as a result of my using health/therapy apps, information about me that I consider private is now more readily available to others than I would want.“] | I believe that as a result of my using mobile apps, information about me that I consider private is now more readily available to others than I would want. |
| Durch meine Nutzung von Gesundheits-/Therapie-Apps erfahren andere mehr über mich, als ich möchte.  [“I am concerned that health/therapy apps may monitor my activities on my mobile device.“] | I am concerned that mobile apps may monitor my activities on my mobile device. |
| Ich glaube, dass durch die Nutzung von Gesundheits-/Therapie-Apps Informationen verfügbar sind, die meine Privatsphäre verletzen können.  [“I feel that as a result of my using health/therapy apps, information about me is out there that can invade my privacy.“] | I feel that as a result of my using mobile apps, information about me is out there that, if used, will invade my privacy. |
| **Secondary use of personal information [35]** | |
| Ich bin besorgt, dass Gesundheits-/Therapie-Apps meine persönlichen Daten ohne meine Zustimmung für andere Zwecke verwenden.  [“I am concerned that health/therapy apps may use my personal information for other purposes without notifying me or getting my authorization.”] | I am concerned that mobile apps may use my personal information for other purposes without notifying me or getting my authorization. |
| Wenn ich persönliche Informationen in Gesundheits-/Therapie-Apps angebe, können diese für andere Zwecke genutzt werden.  [“When I give personal information to health/therapy apps, my information may be used for other purposes.“] | When I give personal information to mobile apps, I am concerned that apps may use my information for other purposes. |
| Ich mache mir Sorgen, dass Gesundheits-/Therapie-Apps meine persönlichen Daten ohne meine Zustimmung an Andere weitergeben.  [“I am concerned that health/therapy apps may share my personal information with other entities without getting my authorization.“] | I am concerned that mobile apps may share my personal information with other entities without getting my authorization. |
