# Supplementary material for: Applying an Extended UTAUT2 Model to Explain User Acceptance of Lifestyle and Therapy Mobile Health Apps: Survey Study
Source: JMIR Mhealth Uhealth. 2022 Jan 18;10(1):e27095. doi: 10.2196/27095 (PMC8808343; doi:10.2196/27095)
Supplement: Multimedia Appendix 2 [file mhealth_v10i1e27095_app2.docx]

**Multimedia Appendix 2: Exploratory Factor Analysis**

To test the validity of the scales with the translated items, we conducted an exploratory factor analysis (following the guidelines of [1]). We used principal axis factor analysis with oblique rotation (direct oblimin). Bartlett’s Test was significant (χ^2^ (630) = 21977.27, p < .001) showing that correlations between items are large enough. The Kaiser-Meyer-Olkin measure verified the sampling adequacy for the analysis (KMO = .93). Factor loadings > .3 were categorized as adequate. The following table displays the factor loadings after rotation with those >.3 in bold, confirming a good solution.

|  | PE | PC | EE | SI | Trust | BI | HM | FC |
| --- | --- | --- | --- | --- | --- | --- | --- | --- |
| PE_1 | **,764** | -,029 | ,037 | -,017 | -,019 | -,082 | -,077 | ,023 |
| PE_2 | **,717** | -,029 | -,050 | ,075 | ,012 | ,051 | -,150 | -,043 |
| PE_3 | **,905** | -,009 | ,019 | ,005 | ,049 | ,029 | ,020 | ,016 |
| PE_4 | **,879** | ,014 | -,015 | -,010 | ,016 | -,052 | ,033 | ,087 |
| PE_5 | **,751** | ,061 | ,073 | ,047 | ,044 | -,066 | -,003 | -,025 |
| EE_1 | ,039 | ,034 | **,789** | -,023 | -,042 | -,066 | ,052 | ,051 |
| EE_2 | -,011 | -,019 | **,906** | ,043 | ,060 | ,030 | -,072 | -,047 |
| EE_3 | -,007 | -,029 | **,940** | ,030 | ,027 | ,034 | -,040 | -,009 |
| EE_4 | ,015 | -,009 | **,902** | ,000 | -,008 | ,003 | -,004 | ,015 |
| SI_1 | ,014 | ,008 | ,052 | **,889** | ,008 | -,045 | ,012 | -,015 |
| SI_2 | ,022 | ,014 | ,020 | **,911** | ,016 | -,007 | -,006 | ,009 |
| SI_3 | ,013 | -,024 | -,026 | **,900** | ,003 | -,037 | -,005 | ,058 |
| FC_1 | ,124 | -,007 | ,035 | -,119 | ,038 | -,096 | ,025 | **,611** |
| FC_2 | ,068 | -,054 | ,293 | -,116 | -,003 | -,043 | -,007 | **,580** |
| FC_3 | ,035 | ,033 | -,006 | ,063 | ,016 | ,100 | -,003 | **,549** |
| FC_4 | -,064 | -,085 | ,076 | ,048 | ,006 | -,052 | -,082 | **,644** |
| HM_1 | ,064 | ,011 | ,060 | -,009 | ,010 | -,104 | **-,813** | ,060 |
| HM_2 | ,175 | -,038 | ,141 | -,023 | ,057 | -,139 | **-,591** | ,017 |
| HM_3 | ,041 | ,029 | -,018 | ,054 | ,062 | -,018 | **-,743** | ,001 |
| BI_1 | ,030 | ,000 | ,007 | ,034 | ,037 | **-,872** | -,056 | -,011 |
| BI_2 | ,044 | ,024 | -,020 | ,066 | ,073 | **-,821** | -,071 | -,034 |
| BI_3 | ,041 | -,019 | ,016 | ,065 | ,039 | **-,853** | -,045 | -,029 |
| Trust_1 | ,049 | -,060 | ,033 | -,047 | **,602** | -,114 | -,013 | ,147 |
| Trust_2 | ,027 | ,015 | ,103 | -,030 | **,652** | -,047 | -,003 | ,096 |
| Trust _3 | ,021 | ,011 | -,031 | ,051 | **,792** | ,087 | -,027 | -,037 |
| Trust_4 | -,008 | -,041 | -,008 | ,008 | **,821** | -,078 | -,005 | -,054 |
| Trust_5 | ,006 | -,010 | -,019 | ,021 | **,847** | -,033 | -,011 | -,047 |
| PC_Surveillance_1 | -,073 | **,364** | ,058 | -,020 | ,054 | -,004 | ,016 | ,231 |
| PC_Surveillance_2 | ,002 | **,812** | ,033 | -,020 | ,026 | ,082 | -,011 | -,032 |
| PC_Surveillance_3 | ,024 | **,841** | -,004 | ,011 | ,028 | ,016 | -,022 | -,084 |
| PC_Intrusion_1 | -,015 | **,844** | -,010 | -,017 | -,046 | ,064 | -,130 | -,016 |
| PC_Intrusion_2 | -,051 | **,788** | -,075 | ,043 | -,038 | -,016 | -,079 | -,026 |
| PC_Intrusion_3 | -,062 | **,823** | -,077 | -,019 | -,041 | -,051 | -,030 | ,036 |
| PC_secondaryuse_1 | ,069 | **,866** | ,018 | ,003 | -,032 | -,030 | ,077 | -,034 |
| PC_secondaryuse_2 | ,006 | **,625** | -,008 | ,013 | -,062 | -,058 | ,075 | ,079 |
| PC_secondaryuse_3 | ,063 | **,866** | ,021 | -,004 | ,014 | ,015 | ,082 | -,103 |
| Rotation converged in 10 iterations.  PE = Performance Expectancy, EE = Effort Expectancy; SI = Social Influence; FC = Facilitating Condition; HM = Hedonic Motivation; BI = Behavioral Intention; PC = Privacy Concerns | | | | | | | | |

**References**

1. Ringle C, Wende S, Becker J-M. SmartPLS. Bönningstedt, Germany: SmartPLS GmbH; 2015. [accessed 2019-01-01]. https://www.smartpls.com/
